# Metabolic control in a nationally representative diabetic elderly sample in Costa Rica: patients at community health centers vs. patients at other health care settings

**DOI:** 10.1186/1472-698X-8-5

**Published:** 2008-05-14

**Authors:** Gilbert Brenes-Camacho, Luis Rosero-Bixby

**Affiliations:** 1 Center for Demography and Ecology, University of Wisconsin-Madison, USA; 2 Centro Centroamericano de Poblacion, University of Costa Rica, Costa Rica

## Abstract

**Background:**

Costa Rica, like other developing countries, is experiencing an increasing burden of chronic conditions such as diabetes mellitus (DM), especially among its elderly population. This article has two goals: (1) to assess the level of metabolic control among the diabetic population age ≥ 60 years old in Costa Rica, and (2) to test whether diabetic elderly patients of community health centers differ from patients in other health care settings in terms of the level of metabolic control.

**Methods:**

Data come from the project CRELES, a nationally representative study of people aged 60 and over in Costa Rica. This article analyzes a subsample of 542 participants in CRELES with self-reported diagnosis of diabetes mellitus. Odds ratios of poor levels of metabolic control at different health care settings are computed using logistic regressions.

**Results:**

Lack of metabolic control among elderly diabetic population in Costa Rica is described as follows: 37% have glycated hemoglobin ≥ 7%; 78% have systolic blood pressure ≥ 130 mmHg; 66% have diastolic blood pressure ≥ 80 mmHg; 48% have triglycerides ≥ 150 mg/dl; 78% have LDL ≥ 100 mg/dl; 70% have HDL ≤ 40 mg/dl. Elevated levels of triglycerides and LDL were higher in patients of community health centers than in patients of other clinical settings. There were no statistical differences in the other metabolic control indicators across health care settings.

**Conclusion:**

Levels of metabolic control among elderly population with DM in Costa Rica are not that different from those observed in industrialized countries. Elevated levels of triglycerides and LDL at community health centers may indicate problems of dyslipidemia treatment among diabetic patients; these problems are not observed in other health care settings. The Costa Rican health care system should address this problem, given that community health centers constitute a means of democratizing access to primary health care to underserved and poor areas.

## Background

Several Latin American countries are advancing quickly through the epidemiologic transition. They are facing a double epidemiologic burden, since the prevalence of chronic illnesses is increasing and some infectious diseases have not been completely eradicated [1-3]. Costa Rica, one of the Latin American leaders in the epidemiologic transition, achieved a very fast reduction in the prevalence of communicable diseases and malnutrition thanks to improved access to primary health care [4]. However, this primary health care system is now struggling with an increase in the aged population and in the burden of chronic and degenerative diseases [2-5]. Adaptation to this new epidemiological profile may be harder in developing countries than in the industrialized world because of the great speed of changes and the relative scarcity of economic resources of the population and of the providers.

Diabetes mellitus (DM) is one of the illnesses with the highest burden among Latin American elderly because survivors of the cohorts born during the first half of the 20^th ^century may be adopting nutritional and lifestyle behaviors of the industrialized world [3,5-7], and at the same time these cohorts experienced disadvantaged health conditions during their younger years [3,8]. These two circumstances are argued to be risk factors for the development of DM [3]. In Costa Rica, the country with the highest life expectancy in Latin America [4], death rates due to DM in 2004 are 2.5 times the rates observed in 1990 [9]. The prevalence of diabetes in 2000 among people 20 years and above was 3.3% in Costa Rica, according to official figures of the World Health Organization (WHO) [10]. It is also the illness with the highest total hospitalization cost, and the second in outpatient costs (after hypertension) for the Costa Rican public hospital and clinic system [11]. Adequate management of DM is becoming an important public health concern in a country whose health care system needs to face the challenge of an increasing burden of chronic diseases.

Studying the burden of chronic diseases in Costa Rica is also becoming important because of its demographic context. The population is aging at a rapid pace. The population age 60 and over will triple from 2000 to 2025, and its share among the total population will increase from 8% to 16%. Costa Rica is also one of the Latin American countries with the highest proportion of the elderly living in rural areas (37%). Regarding ancestry, most Costa Rican elderly can be classified as of "mixed ethnic origins", descendants of a combination of ethnic backgrounds: Europeans, indigenous groups, African blacks, and Chinese immigrants. However, Costa Ricans see themselves as "white" or "mestizo". Around 1% of the people age 60 and above classifies themselves as indigenous and less than 2% as blacks, and these minorities are disproportionately located in the province of Limon, in the Caribbean coast. An important proportion of the black community in Costa Rica descends from immigrants who came to work on the railroad from Jamaica and Barbados during the late part of the 19^th ^century. From a demographic perspective, country of origin is more relevant than race, given that 8% of the elderly population was born abroad, especially in Nicaragua [9].

Although Costa Rica has a mixed health care system, most of the services are provided through a network of hospitals and clinics managed by a public institution-the Social Security system. This system is in part responsible for the high life expectancy levels of Costa Rica [12,13]. The Social Security system is funded from deductions from the payroll, and contributions by the employers and the Government. This is the so-called "contribution regime". Workers that contribute to the System are entitled to health insurance that allows them and their family to receive free health care services and free medications at any hospital, clinic or community health center run by the Social Security system. Additionally, after a "means test", the Government provides health insurance to poor people who are not entitled to it through the "contribution regime". This is the so-called "insurance by the State" or the "non-contribution" regime. Data from the CRELES sample show that the contribution regime covers around 80%; the non-contribution, 15%; and 5% are not insured. There are no copayments for receiving health care. Finally, there are also private hospitals, clinics, and physician offices. People who seek services at these private health care settings have to pay out-of-pocket not only for health care but also for medication and laboratory tests. A new market of private health insurance that caters to high income families is starting to grow in Costa Rica. Interestingly, there are people who use both sectors (e.g., people go for outpatient visits at public clinics but pay out-of-pocket for laboratory examination at private laboratories) [14]. It is important to say also that most of the private hospitals and clinics are located in the Metropolitan Area, where the capital is, while community health centers, and public clinics and hospitals are spread over the Costa Rican territory.

A recent health sector reform, that started around 1995, achieved further advances in reducing child and adult mortality [13]. One of the key elements of the reform was the establishment of community health care centers, called EBAIS (abbreviation in Spanish for "Basic Integrated Health Care Teams"), in different urban and rural settings. The area covered by each EBAIS has an average population of 2,500 to 7,000 inhabitants. These community health centers are staffed with at least a physician, a nurse, and a community health worker; several EBAIS may receive also professional support from an additional team composed of another physician, another nurse, a social worker, a dentist, a nutritionist, a pharmacist, a microbiologist, and a medical records technician [14].

The opening of EBAIS allowed the Government to reorganize the structure of the public health care system. The community health centers are a key component in improving access to primary health care services, since they were first established to serve patients living in rural areas or in poor neighborhoods [13,14]. These community health centers provide outpatient services (preventive services and health promotion), while clinics and hospitals provide primary, secondary and tertiary health care, although they are specialized on the last two types of care: inpatient and more specialized outpatient health care (like surgeries, cancer treatment, intensive care units, or emergencies). Community health centers seek to rationalize resource allocation by referring only those patients who need specialized treatment to specialists in clinics and hospitals. In this sense, they serve as "gate-keepers" for the whole public system. In the past, outpatient visits for chronic disease control were held only in clinics and hospitals. Nowadays, EBAIS have also assumed this kind of consultations as part of their mission of delivering preventive services [14].

Private health care providers deliver the same health care services as the public system, although there is no integration among providers of primary, secondary, and tertiary health care. Besides, private health care services are not organized into health care management programs. Diabetes education and community preventive programs are rare in the private sector, and this is a major difference between both types of providers. The other major difference is the existence of waiting lists in the public system. While patients at public clinical settings have to wait as much as several months (or even years) for an appointment (especially if the appointment is aimed for routine check-ups or laboratory exams), services at private settings are more timely and readily available [14]. Still, a Costa Rican who receives health services at public health centers has gone on average 5.4 times per year to outpatient services, but Costa Ricans that receive services at the private sector have gone on average 3.8 times per year to outpatient visits. The difference is greater among people who have been hospitalized. Among them, the average number of days stayed at a public hospital is 12.6 days and, at a private hospital, it is 5.6 days [15]. In general, access and coverage in the private sector can not be compared to access and coverage in the public sector, since the services of the latter are basically free to all affiliates but affected by long waiting lists, while access and coverage in the private sector depend entirely on purchasing power.

Even though achievements of the Costa Rican health sector reform (which has not yet finished) have been documented [12-14,16], chronic disease management for elderly patients has proved difficult for the public health care system. The system has very high coverage in services aimed to pre-natal care and children (e.g., DPT vaccination = 90%, births delivered in the public system that had pre-natal care = 93%), but only 57% of the elderly population and 84% of the total population with DM received some kind of treatment at any of the public system's primary health care facilities during 2005 [16].

A way of exploring how well community health centers are carrying out the task of providing DM-related preventive services to the elderly is by comparing levels of metabolic control across health care settings. As previously explained, public hospitals and clinics provide outpatient services, but they are aimed to treat cases that need more specialized treatment, while EBAIS are supposed to work as "gate-keepers" providing basic primary health care to patients, before they are remitted to specialists. Patients that receive outpatient services at public hospitals and clinics are expected to be a selected population that needs specialized treatment. A working hypothesis is that, under the current health care system in Costa Rica, patients whose last outpatient visit was at an EBAIS have metabolic control at least as good as patients who had their last outpatient visit at a secondary or tertiary level service (hospitals and clinics). If metabolic control is inferior among community health center patients than among hospital or clinic patients, then this can be interpreted as evidence of limitations in chronic disease management in the public primary health care system.

This article has two goals. The first is to assess the level of metabolic control among the diabetic elderly population in Costa Rica, following the standards proposed by the American Diabetes Association (ADA) [17]. This goal is important for the Costa Rican and Latin American context because there is very little research in the region that measures metabolic control in a nationally representative sample of the diabetic population. The second is to test whether diabetic elderly patients of community health centers have elevated levels of metabolic control that are different from levels of patients from other health care settings in Costa Rica. Metabolic control among diabetic patients is then utilized as an indicator of the efficacy of treatment that the physician prescribes to the patients [17]. The results regarding the second goal will shed light on whether the health sector reform is adequately responding to the increasing burden of highly prevalent chronic diseases among the elderly population.

## Methods

### Data collection

The Costa Rican Study on Longevity and Healthy Aging (CRELES, for its name in Spanish) is an on-going longitudinal study of a nationally representative sample of 2,827 adults born in 1945 or before (ages 60 and over at the first interview) and residing in Costa Rica in the year 2000. The data used in this article refer to the first wave of interviews, conducted from November 2004 through September 2006. This sample size was obtained from a two-step procedure. First, an original sample of 9,600 individuals was randomly selected from the 2000 census database with stratification by 5-year age groups, with oversampling of older individuals. Sampling fractions ranged from 1.1% among those born in 1941–45 to 100% for those born before 1905. In a second step, an in-depth longitudinal study was carried out in a sub-sample of 60 "health areas" (out of 102 for the whole country), selected from the "mother sample" with probability proportional to the population ages 60 and over. This sub-sample included near 4,700 individuals. The sub-sample, which covers 59% of Costa Rican territory, yielded the following non-response rates: 19% deceased by the contact date, 18% not found in the field, 2% moved to other addresses, 2% declined to be interviewed, 2% pendant interviews after several visits (likely rejections). After all these types of non-response are taken into account, the final sample is 2,827 participants. From those interviewed: 95% provided blood sample, 91% had anthropometric measures, and 24% required a proxy to answer the questionnaire.

The in-depth interview addresses topics such as: self-reported health status, functional limitations, household characteristics, utilization of health care services, insurance and working status, inter-generational transfers, and demographic characteristics. All data and specimens in the study were collected at the participants' homes, usually during two visits, using Personal Digital Assistants (PDAs), also known as palm computers, with software applications developed by *Centro Centroamericano de Poblacion *(CCP) for this study. In the first visit, participants provided informed consent for the interview and answered a 90-minute long questionnaire (including some mobility tests and two blood-pressure measures) as well as a 10-minute food frequency questionnaire to trace the intake of specific nutrients. The interviewer also asked permission to check all medication prescribed by a health care professional. In a second visit early the next day, fasting blood samples were collected by venipuncture: 1 EDTA purple top tube (for 3–4 ml. of whole blood) and 2 serum separating tubes (SST) with a clot activator (for 10–12 ml. of blood, to obtain 4–6 ml. of serum). Specimens were analyzed in several laboratories according to the methods described elsewhere [18]. Linear adjustments were performed to have comparable measurements across laboratories; technical support for the adequacy of these linear adjustments was provided by microbiologists. LDL levels were computed using the Friedewald equation [19]. DBP and SBP were computed as the mean of two independent measurements of blood pressure taken during the interview at rest position, with a planned interval of 20 minutes from one another.

### Population and dependent variable definition

The subclass of elderly patients with DM is defined as respondents that answer yes to the question: "Has a doctor or medical personnel ever told you that you have diabetes or high blood sugar levels?" With this question, it is impossible to determine whether the patients have Type I or Type II DM. However, all respondents were diagnosed after age 30, and have survived until at least age 60; therefore, it is likely that most if not all of them have Type II DM. The size of the subsample based on the answer to the above question is 542 individuals, 503 of them with blood sample and valid data for assessing their metabolic control. Since this paper focuses only on people diagnosed with DM, the analyses in this article are performed on this subsample.

Metabolic control is studied by recording levels in glycated hemoglobin (HbA_1C_), diastolic and systolic blood pressure (DBP and SBP), low-density lipoprotein (LDL) cholesterol, high-density lipoprotein (HDL) cholesterol, and triglycerides. Elevated levels of each biomarker are defined according to the standards recommended by the ADA; therefore, elevated levels are: HbA_1C _≥ 7%, DBP ≥ 130 mmHg, SBP ≥ 80 mmHg, LDL ≥ 100 mg/dl, HDL ≤ 40 mg/dl, and triglycerides ≥ 150 mg/dl. High blood pressure is defined when DBP ≥ 130 mmHg and SBP ≥ 80 mmHg [17].

Three more response variables that refer to preventive practices are analyzed: regular aspirin intake, having been vaccinated against influenza during the last year, and having visited a physician at least once in the past year for DM control. Information for these variables is taken from direct answers to questions in the survey instrument. Proportions calculated from the two response variables just listed above are considered as indicators of problems in DM quality care, based on standards of the National Diabetes Quality Improvement Alliance measurement set [20].

### Independent variable definition

The main independent variable is derived from self-reported answers to the question: "Where did you have your last outpatient visit?" Since very few observations were in the categories "private hospital", "private clinic", and "private physician office", these were merged into the single category "private clinical setting."

Potentially confounding variables are derived also from direct answers to the questionnaire by respondents or their proxy respondents: sex, age, income, years of education, region of residence, having health insurance, history of stroke, history of heart attack, smoking, and years since DM diagnosis. Obesity, another potential confounder, is defined as having a body mass index (BMI) higher than 30. BMI was estimated using information on weight in kilograms (kg) divided by height squared in squared meters (m^2^). Weight and height were measured by the interviewers usually during the second visit with an estadiometer (214 Seca Road Rod, Quickmedical) for height and a scale (LifeFource, model UC321P, A&D medical) for weight brought by the interviewer. Standard procedures are used by every interviewer to minimize or avoid measurement bias. Analyses also control for caloric intake, which is estimated from answers to a questionnaire on frequency of consumption of about 30 food-tracer items.

### Statistical analysis

Statistical analysis is conducted using STATA SE version 9.0 (Stata Corporation, 2006). Descriptive statistics include proportions for categorical variables, and means and standard deviations for continuous variables. Ninety-five percent confidence intervals are reported for univariate descriptive statistics. Using logistic regressions to control for confounders, the effect of health care settings on metabolic control are analyzed computing odds ratios. All statistical analyses take into account differential sampling weights.

### Ethics clearance

The University of Costa Rica Ethics Committee approved the procedure for collecting data and the informed consent form. Institutional Review Boards at the University of California-Berkeley and at the University of Wisconsin-Madison reviewed the approval so members of these Universities could participate in the project.

## Results

### Descriptives

Metabolic control may depend not only on health seeking behaviors and treatment compliance by diabetic patients, but also on DM risk factors. According to Table 1, the diabetic population age 60 and above in Costa Rica has a relatively low prevalence of some of these risk factors. Obesity among Costa Rican elderly with DM is around 34%; merely 6% are currently smoking. Only 6% have had a stroke before the interview, and 8% have had a heart attack. Regarding characteristics related with access to health care services, health insurance coverage is almost universal for the Costa Rican elderly: 96% report having health insurance. This high health insurance coverage contrasts with the low socio-economic status (SES) that these cohorts have, given that more than half of older persons in Costa Rica have less than 6 years of education and more than a third of this population have a monthly income of $100 or less (its own if not married, or the average of the couple's monthly income if married).

**Table 1:** Characteristics of the diabetic population age ≥ 60, Costa Rica, 2004–2006. (n = 542)

Characteristics	%
Prevalence of self-reported DM diagnosis	20.8
% Females	61.2
% age 60–69 y.o.	54.0
% age 70–79 y.o.	35.8
% age ≥ 80 y.o.	10.3
% w/6 years or more of schooling	46.8
% w/couple's or individual's income < $100 per month	35.4
% living in Metropolitan capital city	54.0
% w/health insurance	95.7
% w/history of stroke	5.7
% w/history of heart attack	7.9
% w/body mass index (BMI) ≥ 30 Kg/m2	33.6
% currently smoking	6.1
% w/oral medication for DM	69.4
% w/insulin injections	31.0
% diagnosed 0–4 years ago	32.5
% diagnosed 5–9 years	18.2
% diagnosed 10 or more years	47.3
% with year of diagnosis unknown	2.0

As mentioned before, health care in Costa Rica is provided predominantly by the public network of hospitals, clinics, and community health centers. As illustrated in Table 2, 81% of the diabetic population report having their last outpatient visit at a public health care setting. Additionally, although 4% of elderly diabetic patients report to have their last outpatient visit at home, it is very likely that some of them were attended by health professionals from EBAIS, as part of their community home visits.

**Table 2:** Place of last outpatient visit among diabetic population age ≥ 60 in Costa Rica, 2004–2006 (n = 542).

	%
Total	100.0
Public hospital	23.8
Public clinic	23.8
EBAIS	33.4
Private hospital, clinic or physician office	13.2
Patient's home	4.0
Other	1.6
Unknown	0.2

### Levels of metabolic control

Mean levels of biomarkers used as indicators of metabolic control are very close to the endpoints defined as standards by the ADA, which signals that an important proportion of the diabetic elderly population in Costa Rica is not having proper metabolic control. As seen from Table 3, glycemic control, as measured by HbA_1C _levels, seems to be inadequate among 37% of the diabetic elderly. Most also have elevated blood pressure levels. Also indicating high risk, 78% have LDL cholesterol levels above or equal to 100 mg/dl, and 70% have HDL cholesterol levels below or equal to 40 mg/dl. The proportion with elevated triglyceride levels (48%) is slightly lower.

**Table 3:** Levels of metabolic control biomarkers for diabetic population age ≥ 60, Costa Rica, 2004–2006.

Metabolic control indicators	(n)			95% CI	
		Mean	(SD)		

HbA_1C _(%)	503	7.0	(1.8)	(6.8-	7.2)
SBP^a ^(mmHg)	539	148.2	(23.4)	(146.0-	150.4)
DBP^a ^(mmHg)	539	84.6	(12.4)	(83.4-	85.8)
Triglycerides (mg/dl)	516	179.9	(110.0)	(169.4-	190.4)
LDL (mg/dl)	463	131.1	(39.5)	(126.8-	135.4)
HDL (mg/dl)	516	41.2	(11.7)	(40.0-	42.4)

		(%)			

HbA_1C _≥ 7%	503	36.9		(31.8-	41.9)
HbA_1C _≥ 6.5%	503	52.1		(46.9-	57.3)
SBP ≥ 130 mmHg^a^	539	78.2		(74.0-	82.4)
DBP ≥ 80 mmHg^a^	539	65.9		(61.2-	70.6)
SBP ≥ 130 mmHg & DBP ≥ 80 mmHg^a^	539	61.7		(56.8-	66.5)
Triglycerides ≥ 150 mg/dl	495	47.5		(42.3-	52.8)
LDL ≥ 100 mg/dl	463	78.3		(73.8-	82.8)
HDL ≤ 40 mg/dl	516	70.2		(65.5-	75.0)
					
Without aspirin intake	525	39.9		(34.9-	44.8)
Without influenza vaccine last year	537	45.9		(40.8-	51.0)
Without physician examination at least once a year	537	10.2		(7.1-	13.3)

Note: a/Arithmetic mean of two measurements

Regarding preventive behaviors, more than half of the diabetic elderly in Costa Rica are taking aspirin to prevent cardiovascular events, and also less than half were vaccinated against influenza during the last year. There seems to be close contact between diabetic patients and their physicians, given that only 10% lack a diabetes-related examination by their physician at least once a year. Note that sample sizes are uneven across metabolic control indicators, because there were some blood specimens with problems for computing levels of HbA_1C_, Triglycerdies, LDL, and HDL.

### Variation in the standards for HbA1C

Given that the target population in this analysis is comprised of elderly Costa Ricans with DM, it is important to acknowledge that ADA standards might be too strict or aggressive as clinical goals, that higher levels of HbA1C and blood pressure might not be harmful and might even be beneficial for elderly diabetic patients, and that reduction in these biomarker levels might be more important in improving a patient's health than just the absolute target [21-25]. Raising the cutoff points diminishes the prevalence of poor control considerably Table (4). If HbA_1C _≥ 9% defines poor glycemic control, instead of HbA_1C _≥ 7%, prevalence of poor glycemic control decreases in more than half (15% vs 37%). Similar reductions are observed if elevated SBP is defined as SBP ≥ 150 mmHg instead of SBP ≥ 130 mmHg (42% vs. 78%), and if DBP is defined as DBP ≥ 90 mmHg instead of DBP ≥ 80 mmHg (33% vs. 66%). The proportion of diabetic elderly with elevated HbA_1C _and DBP levels is significantly lower (p < 0.05) among people age 75 or more than among people ages 60 to 74. These figures match with previous conclusions that most diabetic elderly in Costa Rica have adequate glycemic control, but are very likely to have high blood pressure levels. Multivariate models in the next section are also estimated using these different cutoff points, but results are not presented in tables unless the conclusions are different from analyses using ADA standards.

**Table 4:** Proportion with poor levels in metabolic control, by age, using different endpoints.

Metabolic control indicators		Age	
	Total	60–74	75 or more

HbA_1C_			
≥ 7%	36.9	39.4	28.9
≥ 8%	21.8	24.0	15.1
≥ 9%	14.8	17.0	7.8
			
SBP			
≥ 130 mmHg	78.2	77.6	79.9
≥ 140 mmHg	62.2	61.1	65.7
≥ 150 mmHg	41.8	41.1	44.0
			
DBP			
≥ 80 mmHg	65.9	69.4	55.1
≥ 85 mmHg	50.9	54.2	40.8
≥ 90 mmHg	33.0	36.4	22.6

We do not present analyses with different endpoints for lipid control because the ADA standards are also recommended by the National Cholesterol Education Program Adult Treatment Panel III [26], given that older persons with DM are considered at high risk of cardiovascular disease. Therefore, ADA standards are not considered as too aggressive.

### Differences in metabolic control across health care settings

In bivariate analyses that do not control for confounders, based on χ^2 ^likelihood-ratio tests of homogeneity, different health care settings show significant differences in just two control indicators: triglycerides (p < 0.01) and lack of annual physician examination (p < 0.10) (Table 5). Regarding the latter condition, patients that had their last outpatient visit at a private clinical setting or at their own home are less likely to have such periodic visits. However, in terms of triglyceride control, elderly patients who had their last visit at a community health center or at home are more likely to have triglycerides ≥ 150 mg/dl. The difference is observed even among patients with prescribed HMG-CoA reductase inhibitors (statins, the most common medication for lipid control observed in the sample). This result contrasts with what is observed with HbA_1C_, hypertension, HDL, or LDL control, since proportions with poor levels in these biomarkers are very similar across health care settings with no statistically significant differences. The absence of significant differences persists even after observing patients with different patterns of treatment, as observed in Table 5, among people prescribed with insulin, with oral hypoglycemic medication, or with antihypertensive medication.

**Table 5:** Proportion with poor levels in metabolic control, by place of last outpatient visit.

Metabolic control indicators	Public hospital (%)	Public clinic (%)	Comm. health centers (%)	Private clinical setting (%)	Patient's home (%)	χ^2 ^p-value
HbA_1C _≥ 7%	36.5	33.6	38.4	43.0	28.9	
-People with insulin (n = 154)	53.3	47.4	68.6	54.5	42.3	
-People with oral medication (n = 346)	38.5	29.3	35.9	47.0	32.0	
						
SBP ≥ 130 mmHg^a^	82.7	74.8	78.3	80.2	68.5	
-People with antihypertensive medication (n = 358)	81.1	73.2	84.3	75.3	72.0	
DBP ≥ 80 mmHg^a^	66.2	66.1	66.0	70.0	50.0	
-People with antihypertensive medication (n = 358)	63.4	60.3	66.8	74.1	52.1	
SBP ≥ 130 & DBP ≥ 80 mmHg^a^	64.4	63.3	60.1	62.1	48.8	
-People with antihypertensive medication (n = 358)	61.8	58.8	65.2	65.9	52.1	
						
Triglycerides ≥ 150 mg/dl	42.5	39.9	56.6	33.6	63.6	***
-People with statin medication (n = 126)	49.6	40.3	71.3	32.0	73.6	**
						
LDL ≥ 100 mg/dl	72.7	79.9	84.0	66.8	70.1	
-People with statin medication (n = 112)	77.7	79.8	84.3	47.7	84.4	
						
HDL ≤ 40 mg/dl	68.9	75.6	70.1	62.4	62.9	
						
Without aspirin intake	33.9	43.9	39.1	46.6	35.8	
Without influenza vaccine last year	43.0	49.1	42.7	59.7	40.8	
Without physician examination at least once a year	13.3	5.6	8.4	19.5	19.4	*

Notes: *: p < 0.10, **: p < 0.05, ***: p < 0.01

a/Arithmetic mean of two measurements

In order to test whether there is confounding effect in these bivariate relationships, one logistic regression for each metabolic control indicator was estimated. Patients who had their last outpatient visit at home are excluded from the analyses because their corresponding sample size is very small (20 persons or less, depending on the indicator). Odds ratios for poor metabolic control levels estimated with the regression models are net of the effect of the characteristics described in Table 1. Controlling for age-one of the variables in the analysis-is important because clinical goals for metabolic control can vary according to patients' age.

Results, shown in Table 6, confirm what was observed previously. There are no significant differences in the prevalence of poor levels of HbA_1C_, SBP, DBP, BP, and HDL, at the community health center when compared to the other health care settings. Results are robust to different patterns of medication therapy, although poor glycemic control is higher in community health center patients than in public clinics and hospitals, when only patients treated with insulin are analyzed (p < 0.10). Elderly diabetic patients treated at public clinics (but not at public hospitals) and at private clinical settings are less likely to have triglycerides ≥ 150 mg/dl, than patients treated at community health centers. The multivariate analyses also show that poor levels of LDL cholesterol are also less common among patients of public hospitals and private outpatient services, than at community health centers. These differences across health care settings seem to be larger when only patients with prescribed statin medication are observed, although the sample size is too small to have an acceptable level of statistical power (more than 80%). Interestingly, among people that visit private clinical settings, frequency of having a DM related examination by a physician seems to be inadequate since they do not report to have at least one physician examination per year.

**Table 6:** Odds ratios for poor levels of metabolic control by place of last outpatient visit.

Metabolic control indicators	(n)	Community health centers	Public clinic		Public hospital		Private clinical setting	
HbA_1C _≥ 7%	460	1.00	0.93		0.84		1.40	
-People with insulin	134	1.00	0.32	*	0.34	*	0.25	
-People with oral medication	321	1.00	0.75		1.14		2.03	
								
SBP ≥ 130 mg/dl^b^	479	1.00	0.82		1.28		1.22	
-People with antihypertensive medication	321	1.00	0.51		0.80		0.43	
								
DBP ≥ 80 mg/dl^b^	479	1.00	1.21		1.11		1.65	
-People with antihypertensive medication	321	1.00	0.83		1.02		1.47	
SBP ≥ 130 & DBP ≥ 80 mmHg^b^	479	1.00	1.34		1.33		1.30	
-People with antihypertensive medication	321	1.00	0.86		1.01		1.02	
								
Triglycerides≥ 150 mg/dl	452	1.00	0.51	**	0.63		0.40	**
-People with statin medication	99	1.00	0.38		0.32		0.17	*
								
LDL ≥ 100 mg/dl	423	1.00	0.72		0.40	**	0.27	***
-People with statin medication	76	1.00	0.15		0.20		0.01	***
								
HDL ≤ 40 mg/dl	472	1.00	1.63		1.17		0.85	
								
Without aspirin intake	471	1.00	1.05		0.80		1.10	
Without influenza vaccine last year	478	1.00	1.40		1.17		1.62	
Without physician examination at least once a year	478	1.00	0.95		1.09		7.10	**

Notes: a/Odds ratios computed from logistic regressions that control for sex, age, income, schooling, region of residence, health insurance, history of stroke, history of heart attack, smoking, obesity, and years since diagnosis of DM. The odds ratios for biomarkers are also controlled for estimated caloric intake. Analyses exclude interviewees who had their last outpatient visit at home, because sample size for that category is too small.

b/Arithmetic mean of two measurements

Another conclusion drawn from the multivariate models (results not shown in the tables) is that DM duration is associated only with elevated levels of HbA_1C _and lack of physician examination during the last 12 months. People who were diagnosed more than 4 years ago are twice as likely to have HbA_1C _≥ 7% as people with more recent diagnosis. On the other hand, patients with more recent diagnosis are also more likely to lack a DM-related physician examination during the last 12 months. Whether this latter finding is due to the problem of waiting lists in the public health care system is not clear.

Why are community health centers having higher proportions of diabetic elderly patients with high triglycerides and LDL levels? The survey instrument has questions about when was the last time respondents have their cholesterol levels measured. Besides, as mentioned before, the questionnaire recorded all prescribed medication that respondents were taking. We thus have data to test whether elevated levels in triglycerides and LDL in EBAIS patients come from lack of periodic cholesterol examination, from differences in type of medication, or from both (Table 7). There are statistically significant differences in the proportions of diabetic elderly patients having their last cholesterol level measured during the last 6 months across health care settings (χ^2 ^likelihood-ratio homogeneity test, p < 0.05). However, people who attended community health centers are more likely to have this test, especially when compared to people at private clinical settings. Finally, there are no statistical differences in the proportions having HMG-CoA reductase inhibitors (statin) medication. Adding these variables in the logistic regressions does not modify the size of the estimated odds ratios reported before; estimating models with other nutritional variables, such as carbohydrate intake or fat consumption or oil used for cooking, or accounting for hypertensive medication (which can alter lipids laboratory results) do not change the size of the odds ratios either (results not shown). Therefore, higher prevalence of elevated triglyceride and LDL levels among community health center patients does not seem to be explained by differences in medication or in frequency of periodic cholesterol-related examination. Given that estimated logistic regressions control for caloric intake, smoking, age, sex, obesity, region of residence, education, income, and DM medication (insulin and oral medication intake), it is unlikely that differences in distributions of this kind of characteristics are the ones that are explaining the association between triglyceride and LDL levels, and health care settings.

**Table 7:** Proportion reporting cholesterol treatment, by place of last outpatient visit.

Lipid control indicators	Public hospital	Public clinic	Community health center	Private clinical setting	χ^2 ^p-value
Cholesterol test in last 6 months	86.8	88.6	94.9	77.7	**
Statin medication (current use)	20.1	22.8	24.1	28.8	

Notes: *: p < 0.10, **: p < 0.05, ***: p < 0.01

Given the concern posed above about how ADA standards might be too burdensome or strict as clinical goals for diabetic older patients, the logistic models of this section are also estimated using the different cutoff points shown in Table 3 for HbA_1C_, SBP, and DBP. Compared to the original models with ADA standards, the size of the odds ratios change, but the same conclusions are drawn: there are no differences in the prevalence of poor levels of HbA_1C_, SBP, and DBP across health care settings (results are not presented but can be provided upon request).

## Discussion

The first goal of this article was to assess the levels of metabolic control among elderly diabetic population in Costa Rica, using the guidelines of the ADA as a point of comparison [17]. Mean levels of HbA_1C_, HDL, and LDL, and the proportion of patients having elevated levels in the same biomarkers, seem high in a first look. However, these are similar or even lower than those reported in high-income populations in the U.S. [20,27-31], Germany [32], Sweden [33], and France [34]. Elevated levels of DBP and SBP are considerably higher than in the U.S., while mean levels of triglycerides are lower [20]. When compared to another developing country, prevalence of poor levels of HbA_1C_, DBP, triglycerides, and LDL are lower in this Costa Rican population than in a slightly younger population in Medellin, Colombia; only the proportion of HDL ≤ 40 mg/dl was higher in Costa Rica than in Medellin [35]. Preventive health behaviors are broadly practiced among the Costa Rican older population with DM: only 40% is not having aspirin therapy regularly, and 46% report not to have an influenza vaccine during the last 12 months. Additionally, only 10% report not to have had a diabetes examination by a physician during the last year.

If less strict endpoints of HbA_1C _are used (8% or 9% instead of 7%), the proportion of diabetic older patients with poor glycemic control decreases considerably, and this change strengthens the argument that, among Costa Rican elderly, levels of metabolic control seem favorable.

Elevated BP levels among elderly Costa Ricans with DM found in this study signals an important public health problem in the country. The combination of hypertension and DM yields very high total costs for the public health care services [11]. Further research is needed to learn the reasons for this poor hypertension control in Costa Rica, especially because there are no differences across health care settings. Preventive behaviors linked more strongly to hypertension than to DM or to dyslipidemias should be studied. Prevalence of high blood pressure in the general elderly Costa Rican population is extremely high: 64% among females and 55% among males [35]. DM patients do not escape to this pattern. Given that cardiovascular disease mortality is not high in this population, one wonders whether blood pressure distribution of other populations is applicable to Costa Rica [37]. High prevalence of elevated blood pressure persists even if cutoffs for defining elevated SBP and DBP are raised to 150 mmHg and 90 mmHg respectively.

Aside from hypertension, Costa Rican elderly with DM do not seem to perform worse at metabolic control than populations in the industrialized world. Why are metabolic control levels so favorable among this population? One possible explanation is that Costa Ricans are commonly inclined to preventive health behaviors. Palmer [38] documents that Costa Rica was, in 1914, one of the few Latin American countries in which the goals of the Rockefeller Foundation anti-hookworm campaign were achieved. According to Palmer, the success of this campaign can be explained by the active involvement of Government employees and rural teachers, and the willingness of the Costa Rican population to trust in these public workers. Regarding more recent data, with a non-representative sample of diabetic patients in San Jose, Costa Rica's capital, Firestone et al. found that the levels of DM-specific knowledge were greater than in a sample of Spanish-speaking U.S. Latinos in Starr County, Texas [39].

However, in general, achievements in health status in Costa Rica have been linked to effective primary health care services, provided mainly by public institutions and with a strong commitment with equity [12-14]. Although the Costa Rican government had a long tradition of providing those services (which was in part possible because of the non-existence of military expenditures since the 1949 Constitution abolished the armed forces), in the 1970s there was a breakthrough with the opening of hundreds of Rural Health Posts [4]. More recently, a health sector reform launched in 1995 fostered the opening of community health centers or EBAIS to improve the access to primary health care services in underserved areas. It is hard to tell whether this "Costa Rican model" can be exported to other Latin American countries with much larger or less integrated populations, weaker institutions and fewer resources available for public health.

These free primary health care services have been key mechanisms in the control of communicable diseases, maternal mortality, and children malnutrition. Nonetheless, the epidemiologic transition accompanied with the process of population aging [2,3] can bring changes that occur faster than the institutional ability to respond to them. This possible lag in institutional response may undermine the efficacy of this kind of services, especially in chronic disease management.

The article's first goal-the assessment of metabolic control among the older population in Costa Rica-serves as an introduction for the second goal: to study whether metabolic control among EBAIS patients is different than metabolic control among patients at other health care settings. The working hypothesis was that EBAIS older patients with DM had metabolic control levels equal or better than diabetic elderly patients at other clinical settings because EBAIS are aimed to provide only primary health care services, and patients who need specialists are then referred to physicians at hospitals or clinics. The present article shows that, among the elderly population with DM in Costa Rica, there are no major differences in poor metabolic control between patients from public hospitals and clinics on one side, and patients from community health centers on the other. However, community health center patients are more likely to have elevated levels of LDL and triglycerides. Since worse levels of metabolic control are not found for the other studied biomarkers (HbA_1C_, HDL, SBP, and DBP), these results may mean that lipid control for elderly diabetic patients at community health care centers is not as thoroughly examined as at public hospitals and clinics, or at private clinical settings, perhaps because many EBAIS lack laboratory facilities. No differences in lipid-control medication intake or frequency of cholesterol examination were found across clinical settings; besides, differences in triglycerides and LDL levels are not explained by differences in obesity or caloric intake, given that the multivariate models control for the effect of these variables. These differences may also be related to particularities in DM education at community health centers.

It is important for the public health care system to address the high levels of blood pressure as well as the poorer lipid control among patients that go to EBAIS because these community health centers aim to improve the access to primary health care to underserved and poor populations, and primary health care is an effective way of managing chronic diseases such as DM by decreasing the need of secondary and tertiary health care services [40,41]. Even in industrialized countries such as the U.S.A., community health centers have been seen as an inexpensive way of providing preventive services to underserved and poor populations [31,42,43]. However, in terms of DM control, community health centers in other countries have not always been successful [30,31,43].

The advanced stage of the epidemiological transition in Costa Rica is happening in the context of a health sector reform. The community health centers, a key component of the reform, are increasingly providing primary health care services to elderly patients with chronic disease. If the system of community health centers in Costa Rica turns to be successful in reducing the burden of both communicable and chronic diseases, the Costa Rican health sector reform can serve as an experiment for other health sector reforms that international organisms are fostering in developing and transitional countries [45].

It is also worth noting that there are no differences in metabolic control between patients treated at public services and patients treated at private services, except in the frequency of DM examination by physicians. Diabetic elderly patients that visited a private outpatient service or that were treated at their own house are more likely to lack physician examination at least once a year. It is not clear whether this difference is due to financial barriers or medical practices, but the results should call the attention to users and providers of private health care in Costa Rica.

The analysis in this paper is performed using data from a nationally representative survey of elderly in Costa Rica that gathers self-reported information, as well as biomarkers. This kind of dataset is rare in developing countries. However, there are limitations in the data. An important one is that the study relies on self-reported information which might be affected by reporting bias. Diabetic respondents in the survey were determined based on self-reported answers to the question "Has a doctor or medical personnel ever told you that you have diabetes or high blood sugar levels?" This approach might overestimate the size of the actual diabetic population, and introduce biases in summarizing the outcome variables. If a physician ever told a person that she had impaired glucose tolerance or a one-time elevation in glucose, it is possible that this non-diabetic person will answer "yes" to the question in the survey instrument. We consider that it is inconvenient to validate the self-reported diagnosis with metabolic data because metabolic data is the outcome of this article. Therefore, any correction based on the biomarkers will bias upwards the prevalence of poor metabolic control. A way to explore the bias due to self-report is by using complementary information. The survey inquires about insulin use and other prescribed medication that respondents have in their house. Interviewers ask to see all prescribed drugs and enumerate them. Thirteen percent of the sample (weighted estimate) report not to be taking insulin or oral medication and also lack diabetes-related medication in their houses. If excluding this group, results are roughly the same although the mean levels of the biomarkers and the prevalence of poor metabolic control are slightly higher than with the original sample, but the differences are not statistically significant (α = 0.10). The most important differences between the two sets of results are that the difference in prevalence of LDL ≥ 100 mg/dl between community health center patients and patients at other health centers widens, especially among patients with statin medication, and that the percentage of elderly diabetic people without physician examination during the last 12 months drops from 10% to 4%. We decided not to exclude respondents without DM medication from the analyses because, even though some of them might be persons that do not have the disease, others might be diabetic patients that do not care about the treatment for their illness. Therefore, the analysis would be based on a selected population of people that do care about controlling their disease. We do not report the results based on this subsample that excludes these potential non-diabetic respondents, but we can provide them upon request.

The limitation due to self-reported data is also true for the main explanatory variable, place of last outpatient visit, as well as for most of the control variables. Reporting bias may arise from people that identify community health centers as clinics. Associated with this limitation, there is the problem that the survey questionnaire asks for the place of the last outpatient visit, but the questionnaire inquires neither whether the person visited another health care setting before the last outpatient visit, nor the reason of the last outpatient visit. It is possible that an undetermined proportion of the diabetic older population that reported last outpatient visits might refer to consultations about health problems other than DM-related. We believe this lack of information would not introduce a strong bias in the estimates, insofar as physicians are supposed to be examining patients' DM control, regardless of the reason for the visit. However, this kind of bias can not be discarded.

Another data problem is its limited statistical power to detect some differences, because the sample size of all DM patients is just about 500. Given that the subsample size of EBAIS patients is around 180, and the levels of metabolic control at the EBAIS range from 38% to 84% (depending on the indicator of metabolic control), the logistic regressions estimated for the final analyses have a statistical power of 0.80 to detect only odds ratios that are larger than 1.75 (or smaller than 0.55) for most of the biomarkers, and larger than 2.50 (or smaller than 0.30) for annual physician examination. Statistical power decreases even more when subsamples based on medication therapy are analyzed rather than the whole diabetic subsample. Power calculations are based on formulas for logistic regression analysis [46].

## Conclusion

Levels of metabolic control among elderly population with DM in Costa Rica are similar or better than levels observed in industrialized countries. Nevertheless, unusually high levels of BP indicate a problem in hypertension treatment among diabetic population age ≥ 60 years old. High BP levels are similar across health care settings. The study posed the hypothesis that poor metabolic control was less likely to occur among community health center patients than among patients from other clinical settings. Prevalence of elevated levels of SBP, DBP, and HbA_1C _is very similar across health care settings. Higher proportions of people with elevated levels of triglycerides and LDL at community health centers may indicate problems of dyslipidemia treatment that are not observed in other health care settings. These higher proportions do not appear to be explained by differences in prescribed medication, frequency of lipid control by a physician, obesity, or caloric intake. The establishment of community health centers has been a key component of the health sector reform carried out since 1995 in Costa Rica. Eliminating these differences in metabolic control across health care settings is important for the health care system in the country because community health centers constitute a means of democratizing access to primary health care to underserved and poor areas, and because DM produces high financial costs to the national health care system.

## Competing interests

The authors declare that they have no competing interests.

## Authors' contributions

GBC proposed the article's objectives, carried out the statistical analysis, and wrote the draft manuscript. LRB conceived the CRELES study, directed its design, data collection and data processing, managed it as PI, and supervised the analyses. All authors read and approved the final manuscript.

## Pre-publication history

The pre-publication history for this paper can be accessed here:


